# Referral to cancer genetic counseling: do migrant status and patients’ educational background matter?

**DOI:** 10.1007/s12687-017-0326-4

**Published:** 2017-09-04

**Authors:** J. A. M. van der Giessen, E. van Riel, M. E. Velthuizen, A. M. van Dulmen, M. G. E. M. Ausems

**Affiliations:** 1 Department of Genetics, Division of Biomedical Genetics, University Medical Center Utrecht, PO Box 85090, Utrecht, 3508 AB The Netherlands; 2 NIVEL (Netherlands Institute for Health Services Research), Utrecht, The Netherlands; 3 Department of Primary and Community Care, Radboud University Medical Center, Nijmegen, the Netherlands; 4 Faculty of Health Sciences, University College of Southeast Norway, Drammen, Norway

**Keywords:** Cancer genetic counseling, Referral, Migrant status, Educational level

## Abstract

Participation rates in cancer genetic counseling differ among populations, as patients with a lower educational background and migrant patients seem to have poorer access to it. We conducted a study to determine the present-day educational level and migrant status of counselees referred to cancer genetic counseling. We assessed personal characteristics and demographics of 731 newly referred counselees. Descriptive statistics were used to describe these characteristics. The results show that about 40% of the counselees had a high educational level and 89% were Dutch natives. Compared to the Dutch population, we found a significant difference in educational level (*p* = < 0.01) and migrant status (*p* = < 0.001). This suggests disparities in cancer genetic counseling and as a result of that, suboptimal care for vulnerable groups. Limited health literacy is likely to pose a particular challenge to cancer genetic counseling for counselees with a lower education or a migrant background. Our study points to considerable scope for improvement in referring vulnerable groups of patients for cancer genetic counseling.

**Electronic supplementary material:**

The online version of this article (10.1007/s12687-017-0326-4) contains supplementary material, which is available to authorized users.

## Introduction

For families in which a hereditary form of cancer is suspected, cancer genetic counseling can add to the optimal treatment and clinical management of patients. It can serve as a valuable aid for surveillance and/or preventive surgery for patients affected by cancer and their unaffected family members. Therefore, identifying patients eligible for cancer genetic counseling and referring them is important. Unfortunately, due to various physician- and patient-related factors (Brandt et al. 2008), not all patients eligible for genetic counseling are recognized by their physicians (Kurian et al. 2017).

### Physician-related factors

From physicians’ perspective, patient eligibility for cancer genetic counseling is based on family history, patient cancer history, and patient request (Brandt et al. 2008). The first challenge for physicians is to identify patients eligible for referral by gathering adequate information about the family history. As shown, EMRs (electronic medical records) do not always contain enough information about family history (Sollie et al. 2016; Vogel et al. 2012). Vogel et al. (2012) found that only 50% of the patients who are eligible for referral for Lynch syndrome and hereditary breast and ovarian cancer could be identified by the EMR. Current standard clinical practices seem insufficient at identifying patients who meet the criteria for referral to genetic counseling.

The majority of physicians experience a lack of time to collect detailed information about family history (Al-Habsi et al. 2008; Wood et al. 2008). Insufficient knowledge about hereditary cancer and about the criteria for referral acts as another barrier for referral (Dekker et al. 2013; Panic et al. 2014). Besides, physicians tend to overestimate the risk of patients who actually are at population risk, while underestimating risk of patients who are at increased risk of developing cancer (Baldwin et al. 2014). Despite the fact that referral guidelines are sufficiently available, numerous studies have shown that physicians are still lacking knowledge of genetics and the latest criteria for referral (Douma et al. 2016; Prochniak et al. 2012).

When physicians are convinced, there is a high probability that the risk is hereditary; they have a tendency to refer patients for periodic screening examinations instead of genetic counseling (Burke et al. 2009; Sollie et al. 2016). For colorectal cancer syndromes, Prochniak et al. (2012) found that, although physicians endorsed guidelines as a significant influence on their practice decisions, these guidelines did not influence the referral to cancer genetic counseling. The low adherence to guidelines for referral and poor registration of family history may also be responsible for the differences in referral to cancer genetic counseling in migrant patients. In a study among Turkish and Moroccan patients by Baars et al. (2016), the lowest referral rates are observed in the group of women with breast cancer at young age, despite the fact that age < 40 years is a criterion for referral, independent of family history and migrant status (Baars et al. 2016).

### Patient-related factors

Patient characteristics influence the referral process as well. Patients’ request for cancer genetic counseling and their concerns about family members are important determinants for cancer genetic counseling. Van Riel et al. (2012) showed that the majority of counselees initiate a referral themselves (van Riel et al. 2012), while Brandt et al. (2008) found that 73% of a group of primary care physicians and specialists base patient eligibility for referral on patient request, and 54% did not refer eligible patients due to patient disinterest. When physicians are not sure whether screening is recommended, patients who expect screening and those who are more anxious, are more often referred (Haggerty et al. 2005). Apparently, cancer-related concerns, but also perceived cancer risk and the belief that family history influences cancer risk, contribute to referral for cancer genetic counseling (Bellcross et al. 2015).

Lack of awareness and/or knowledge about personal risk, medical history, and genetic services, seem to act as barriers to referral to cancer genetic counseling (Delikurt et al. 2015). Research from Allford et al. (2014) suggests low awareness and understanding of familial cancer risk among minority ethnic communities (Allford et al. 2014). Also, socio-cultural variations in beliefs, notably stigma about cancer of inherited risk of cancer, have been identified. For migrant breast cancer patients, language difficulties and lower health literacy, as well as cultural factors, are determinants for non-participation in genetic counseling (Baars et al. 2017).

Sharing information with the physician about family history in relation to cancer, is an important factor in the referral process. Patients with a lower social economic status, a lower educational background, or a migrant background, experience greater verbal passivity and difficulties in presenting health-related information to their physician (Cooper and Roter, 2003).

### Differences in cancer incidence

Cancer incidence may vary in the population and therefore result in different participation rates in cancer genetic counseling. Migrants and people of low socioeconomic status (SES) share certain cancer risks, like the lower risks for colon, skin, breast, and prostate cancer (Aarts et al. 2010; Arnold et al. 2010). Non-western migrants exhibit a higher burden of infection-related tumors (Arnold et al. 2010; Dutch Cancer Society 2006; Visser and van Leeuwen, 2007). The difference in cancer incidence in the population is complex, because higher cancer awareness and participation in cancer screening programs, might have contributed to a higher incidence for certain types of cancer, like breast cancer. This is usually promoted more by patients of high SES (Aarts et al. 2010). Recent research from Welch and Fisher (2017) confirms that cancer screening is one area in which overutilization can result in overdiagnosis, particularly for cancers for which the reported incidence is sensitive to early screening programs (Welch and Fisher, 2017). Reported higher incidence does not seem to lead to a parallel increase in prevalence, as shown by the database from the National Institute of Public Health and the Environment (RIVM). This database showed no significant difference in prevalence of cancer between the lowest and highest educated people (National Institute for Public Health and the Environment 2012).

### Cancer genetic counseling

In order to gain more insight in factors associated with referral to genetic counseling, we conducted an observational study in 2007 (van Riel et al. 2012). That study showed that, compared to the general population, more highly educated counselees and less migrant counselees were seen in cancer genetic counseling practice.

Since 2007, more information has become available about hereditary cancer and referral for cancer genetic counseling. For physicians, guidelines have been updated and published about criteria for referral (Balmana et al. 2013; Berliner et al. 2013; Giardiello et al. 2014). Also, additional value of interventions, such as an online referral test, an interactive web-based training and an electronic referral form, has been reported (Bell et al. 2015; Dekker et al. 2014; Petzel et al. 2014).

For the general public, attention to this subject has been drawn in the media, e.g., by the release of a public statement from actress Angelina Jolie (Evans et al. 2014; Roberts and Dusetzina, 2017).

Over the years, for both physicians as the general population, awareness about cancer genetic counseling has increased. This is expected to be reflected in a cancer genetic counseling population which is more comparable to the general population when it comes to educational level and migrant status. To study this expectation, we conducted a study, 8 years after our previous study, with the aim to determine the present-day educational level and migrant status of counselees referred to cancer genetic counseling and to investigate possible differences with our 2007 data.

## Methods

### Participants

Participants were newly referred counselees for cancer genetic counseling from October 2014 to April 2015. Similar to our previous study (van Riel et al. 2012), counselees were seen by a clinical geneticist or genetic counselor from the department of Genetics of the University Medical Center Utrecht at either the university main site or one of the nine community hospitals in the region.

### Study design and data collection

For each new counselee, a checklist was filled in by the counselor during the first consultation. In this checklist, several items were scored: general characteristics of the counselee and the consultation, eligibility for genetic testing, educational level, country of birth of counselee and his/her parents (see Electronic Supplementary Material).

Educational level was determined by the Dutch Standard Classification of Education (Statistics Netherlands 2008) and the international classification of the UNESCO (Unesco Institute for Statistics 2011): low educational level: (pre-)primary education or first stage of basic education; intermediate-1 educational level: lower secondary or second stage of basic education; intermediate-2 educational level: (upper) secondary education; and high educational level: tertiary education.

Migrant status of the counselee was determined according to the definition of Statistics Netherlands (Statistics Netherlands 2008). According to this definition, a counselee is a migrant when at least one of the parents is born outside of the Netherlands. Furthermore, a distinction can be made between Western migrants (at least one parent born outside the Netherlands, but in Europe, North America, Australia, New Zealand, Indonesia, and Japan) and non-Western migrants (at least one parent born in Turkey and countries in Africa, Latin America, and Asian countries). The classifications of educational level and migrant status of Statistics Netherlands was chosen to allow comparison with data about the general population of the Netherlands. Also, these same classifications were used in our earlier study (van Riel et al. 2012), so, comparison with the data of the current study with the situation in 2007 is possible.

Eligibility for genetic testing was determined for the counselee or for an affected family member of the counselee based on family history and/or (if available at initial consultation) medical records, according to national guidelines for different tumor syndromes used in daily practice.

The subgroup “other” in eligibility for genetic testing contains several reasons, e.g., eligibility can be determined after receiving the medical records of the counselee and/or family members, which are not always present at first consultation. Also, in initiating discussion of family history, a category “other” exists. This category contains initiating discussion of family history by a family member, by a family letter (a letter in case a mutation in a cancer gene is detected, intended to share with family), by the counselee and physician together, or by the physician of a family member.

### Statistical analysis

All data were entered in SPSS Version 21.0.0. Descriptive statistics were used to describe counselee characteristics, for university and community hospitals separately, and for both clinics combined. Chi-square tests were used to compare the collected data to the data of the general population in the Netherlands (Statistics Netherlands 2014a; Statistics Netherlands 2014b).

## Results

### General characteristics

In total, 731 counselees were included. General characteristics, like clinical setting of the consultation, gender and personal cancer history of the counselee, and eligibility for genetic testing are shown in Table 1. There were more female counselees compared to male counselees, as more than half (56%) of the affected counselees had breast cancer. About half of all counselees were seen in the university hospital (55%); the other 45% were seen in community hospitals. This is the same distribution as reported earlier in our study in 2007 (van Riel et al. 2012).

**Table 1:** General characteristics of 731 counselees requesting cancer genetic counseling

Variable		Both clinics combined % (*n* = 731)	University hospital % (*n* = 403)^a^	Community hospitals % (*n* = 328)^a^	*p* value
Total		100.0	55.1	44.9	
Gender	Male	25.6 (187)	26.6 (107)	24.4 (80)	n.s.
	Female	74.4 (544)	73.4 (296)	75.6 (248)	
Personal cancer history	Affected	50.8 (371)	46.7 (188)	55.8 (183)	0.014*
	Unaffected	49.2 (360)	53.3 (215)	44.2 (145)	
Affected with (*n* = 371)	Breast cancer	56.3 (209)	60.6 (114)	51.9 (95)	n.s.
	Ovarian cancer	4.0 (15)	2.7 (5)	5.5 (10)	n.s.
	Colon cancer	16.4 (61)	10.1 (19)	23.0 (42)	0.001*
	Endometrial cancer	0.8 (3)	1.6 (3)	0 (0)	n.s.
	Melanoma	2.7 (10)	4.3 (8)	1.1 (2)	n.s.
	Polyposis	9.2 (34)	9.6 (18)	8.7 (16)	n.s.
	≥ 2 kinds of cancer	5.1 (19)	5.3 (10)	4.9 (9)	n.s.
	Other^b^	5.4 (20)	5.9 (11)	4.9 (9)	n.s.
Eligibility for genetic testing in counselee or relative	Diagnostic DNA testing	38.2 (279)	36.0 (145)	40.9 (134)	n.s.
	MSI/IHC^c^	11.6 (85)	9.4 (38)	14.3 (47)	0.040*
	Predictive testing^d^	22.0 (161)	29.5 (119)	12.8 (42)	0.000*
	Did not meet criteria for testing	10.4 (76)	8.7 (35)	12.5 (41)	n.s.
	Other	17.8 (130)	16.4 (66)	19.5 (64)	n.s.
Initiator discussing family history	Counselee	35.6 (252)	38.3 (148)	32.4 (104)	n.s.
(*n* = 707)	Physician	48.4 (342)	42.2 (163)	55.8 (179)	0.000*
	Other	16.0 (113)	19.4 (75)	11.8 (38)	0.006*
Educational level^e^ (*n* = 714)	Low	5.0 (36)	4.1 (16)	6.2 (20)	n.s.
	Intermediate-1	21.6 (154)	18.7 (73)	25.1 (81)	0.038*
	Intermediate-2	35.2 (251)	31.7 (124)	39.3 (127)	0.034*
	High	38.2 (273)	45.5 (178)	29.4 (95)	0.000*
Migrant status^f^ (*n* = 723)	Dutch native	88.7 (641)	86.6 (342)	91.2 (299)	0.053*
	Migrant	11.3 (82)	13.4 (53)	8.8 (29)	
	Migrant, western	6.7 (49)	7.1 (28)	6.4 (21)	n.s.
	Migrant, non-western	4.6 (33)	6.3 (25)	2.4 (8)	0.013*

^*^A two-sided *p* value of < 0.05 is considered significant. n.s.: not significant

^a^Data calculated for clinical setting (i.e., within each column)

^b^Other cancer: parathyroid adenoma, angiolipoma, carcinoid, brain tumor, hyperparathyroidism, pituitary tumor, leiomyomatosis, leukemia, neurofibroma, kidney cancer, pancreatic cancer, prostate cancer, sarcoma, sebaceoma, esophageal cancer, and testis carcinoma

^c^MSI/IHC: microsatellite instability testing/immunohistochemistry for mismatch repair deficiency

^d^Predictive testing: genetic testing for a mutation which is already known in the family of the counselee

^e^Low: (pre-)primary education or first stage of basic education; Intermediate-1: lower secondary or second stage of basic education; Intermediate-2: (upper) secondary education; High: tertiary education

^f^Dutch native: both parents are born in The Netherlands; Migrant: at least one of the parents is born outside the Netherlands. Western Migrant: at least one parent born outside the Netherlands, but in Europe, North America, Australia, New Zealand, Indonesia, and Japan; Non-Western Migrant: at least one parent born in Turkey and countries in Africa, Latin America, and Asian countries

When we compare the referral between university hospital and community hospitals, we found that more counselees affected with colon cancer were seen in community hospitals, which explains the higher eligibility for microsatellite instability testing/immune histochemistry (MSI/IHC) of counselees seen in community hospitals. In the university hospital, more counselees were seen for predictive testing for a known mutation.

In initiating discussion of family history, we found discussion started by a family member in 58%, by a family letter in 23%, by the counselee and physician together in 14%, and by the physician of a family member in 5%. In community hospitals, the physician more often initiated discussion of family history, and less often, this discussion is initiated by “other” (e.g., a family member or via a family letter).

### Educational level

When classified according to the International Standard Classification of Education (Unesco Institute for Statistics 2011), about 40% of the counselees seen for cancer genetic counseling had a high educational level. When compared for clinical setting, more counselees with an intermediate-1 and intermediate-2 level of education were seen in community hospitals, and more highly educated counselees were seen in the university hospital. In comparison with the Dutch population (Table 2), less counselees with a lower and intermediate-2 educational level and more highly educated counselees were seen in cancer genetic counseling. No significant difference was found in the educational level of counselees in 2007 and in 2014/2015 (van Riel et al. 2012) (Table 3).

**Table 2:** Educational level and migrant status of counselees in cancer genetic counseling in comparison to the general population in the Netherlands

	This study (2014/2015) % (*n*)	General population (2014) % (*n*)	*p* value
Educational level			
Low	5.0 (36)	9.8 (1,229,000)	< 0.01
Intermediate-1	21.6 (154)	21.0 (2,625,000)	0.7033
Intermediate-2	35.2 (251)	40.7 (508,900)	< 0.01
High	38.2 (273)	28.5 (3,564,000)	< 0.01
Migrant status			
Dutch native	88.7 (641)	78.6 (13,234,545)	< 0.001
Migrant	11.3 (82)	21.4 (3,594,744)	
- Western	6.8 (49)	9.5 (1,597,160)	
- non-Western	4.6 (33)	11.9 (1,997,584)

**Table 3:** Educational level and migrant status of counselees in cancer genetic counseling in comparison to the study in 2007 (Van Riel et al. 2012)

	This study (2014/2015) % (*n*)	Study data (2007) % (*n*)	*p* value
Educational level			
Low	5.0 (36)	4.0 (16)	0.4340
Intermediate-1	21.6 (154)	26.3 (105)	0.0723
Intermediate-2	35.2 (251)	33.3 (133)	0.5400
High	38.2 (273)	36.3 (145)	0.5314
Migrant status			
Dutch native	88.7 (641)	90.6 (368)	0.2998
Migrant	11.3 (82)	9.4 (38)	

### Migrant status

The majority of counselees seen for cancer genetic counseling were Dutch natives (89%). There is a trend for less migrants seen in the community hospitals (*p* = 0.05). When migrants of Western and non-Western origin were compared, a significantly lower percentage of non-Western migrants is seen in community hospitals than in the university hospital. Furthermore, there were less migrants seen in cancer genetic counseling compared to the general population (Table 2) (*p* < 0.001). We found no significant difference in frequency of migrants referred for cancer genetic counseling 2014/2015 and 2007 (van Riel et al. 2012), (Table 3).

## Discussion

Our findings suggest that patients’ migrant status and educational background seem to matter in the referral to cancer genetic counseling. In 2007, we found an underrepresentation in cancer genetic counseling of migrant patients and patients with a low educational background (van Riel et al. 2012). The results of the current study show that this underrepresentation has not changed since then. This differential access to cancer genetic counseling may lead to treatment and outcome disparities in cancer care.

The differences in educational level and migrant status, seen between counselees in the university hospital and counselees seen in the community hospital, must be taken into account. However, definite conclusions cannot be drawn from these data because of potential differences in the patient population at the different locations. Socio demographic characteristics may have impact on patients’ communicative behavior (e.g., asking questions or expressing concerns) as well on physicians’ behavior (discussing referral possibility) (Baars et al. 2017).

In the last years, more information about cancer genetics and genetic counseling has become accessible to the general public. From popular magazines, there is an increasing focus on hereditary cancer and referral for DNA-testing. This may affect people’s awareness for cancer genetic counseling and may even contribute to more patient request. As we know, patients’ initiative is important in the referral process (Brandt et al. 2008; Wideroff et al. 2003). Asking questions about genetic testing increases the likelihood of being referred for genetic counseling (Al-Habsi et al. 2008; Klitzman et al. 2013). This might be an explanation for the relatively low attendance of migrant patients and patients with a lower educational background. Asking questions is associated with someone’s health literacy skills (Katz et al. 2007). Health literacy skills reflect the ability to access, understand, appraise, and use health-related information in various domains (Sorensen et al. 2012) and are associated with lower patient activation (Smith et al. 2013). People with a lower socio-economic position, a lower educational level, and a lower subjective social status, are known to have lower health literacy skills than those with a high socio-economic position (Heide van der et al. 2013). Also, many migrant patients have lower health literacy skills (Fransen et al. 2013). This might result in low awareness or understanding of familial cancer. In combination with socio-cultural variations in beliefs about cancer, this may affect patient-doctor communication, as well as referral to cancer genetic counseling (Baars et al. 2017). Given the fact that the majority of counselees seem to initiate referral to genetic counseling themselves (Brandt et al. 2008; van Riel et al. 2012), lower health literacy and corresponding lower patient activation, might contribute to the lower referral rate in migrant patients and in patients with a lower level of education. Physicians will have to adapt their communication to this group of patients in order to allow effective communication and get a higher referral rate. Recent research (Kurian et al. 2017) emphasizes the importance of oncologists’ behavior in the genetic testing process. Improving their communication skills and risk estimation and optimizing triage to genetic counselors have priority.

Lower educated counselees may have other needs for genetic care than higher educated counselees (Hayat et al. 2012), who argue for a more personalized approach in both the referral process and in the genetic counseling itself. Culture-sensitive interventions can ameliorate referral to cancer genetic counseling (Hall and Olopade, 2006). Our study points to room for improvement in referring vulnerable groups of patients. Since the outcome of cancer genetic counseling can give reasons to choose another treatment procedure, this is even more important (Christinat and Pagani, 2013; Glenn et al. 2012; Wevers et al. 2012). Limited health literacy is likely to pose a particular challenge to cancer genetic counseling for counselees with a lower education or a migrant background. Future studies can explore how physicians should assess patients’ need and skills and which communication strategies are effective.

## Limitations

Our findings cannot be generalized as the study was conducted in one single clinical genetic center. However, we included a rather large number of counselees (over 700 consecutive counselees seen for cancer genetic counseling) who were seen in several hospitals in the central region of the Netherlands. Due to the study design, we do not have data about counselees who declined referral for cancer genetic counseling. These possible decliners may have had a different educational background. Also, we did not measure the level of health literacy of the patients, but considered educational background as a proxy (Heide van der et al. 2013; Martin et al. 2009). Numerous studies show the importance of patient request or initiative in the referral process. However, in our study, we found no association between initiative of the counselee and participation in cancer genetic counseling. That is probably because of bias in the scoring procedure. Although we asked who took the initiative for referral, we realize that this outcome is fairly unreliable. It is not always clear who took the initiative and sometimes the respondents did not even remember.

The observed difference in referral between university and community hospitals might be influenced by the approach of our genetic clinic: When a pathogenic mutation is identified in the index case, he/she receives a family letter to inform family members. With this letter, family members can directly contact our department at the university hospital, and are more often invited for a consultation at this location. Furthermore, more information about characteristics of the referring physicians and their practice might lead to a better clarification of the differences seen between consultations in the different clinical settings.

As cancer incidence, as well as demographics of the Dutch population, vary over years, this may influence the referral for cancer genetic counseling. In our study, we did not standardize for these differences. Related to migrant status and socioeconomic inequalities, a variety of studies from Europe has shown that disparities in the burden of cancer exist (Arnold et al. 2010). In further research, we must take these differences into account.

To conclude, the results in this study are similar to the results in 2007. Lower participation in cancer genetic counseling by migrant patients and patients with a lower educational background is still a cause for concern. Additional research on interventions on how to improve referral for these patients is urgently desired.

## Electronic supplementary material

ESM 1 (DOCX 14 kb)
