# Supplementary material for: Referral to cancer genetic counseling: do migrant status and patients’ educational background matter?
Source: J Community Genet. 2017 Sep 4;8(4):303–10. doi: 10.1007/s12687-017-0326-4 (PMC5614888; doi:10.1007/s12687-017-0326-4)
Supplement: Supplementary file 1 — (DOCX 14 kb) [file 12687_2017_326_MOESM1_ESM.docx]

**Checklist**

| Clinical setting: ❑ university hospital ❑ community hospital | Date of consultation: |
| --- | --- |
| Research number: | |

**Disease status**: ❑ unaffected ❑ affected: 🡪 ❑ breast cancer

❑ other: ……………………

**Time-span for genetic testing**: ❑ regular

❑ rapid genetic testing

**Reason for referral**: ❑ hereditary breast cancer

❑ Lynch syndrome

❑ other tumor predisposition syndrome: ……………….

**DNA-testing**: ❑ diagnostic DNA-testing

❑ predictive testing

❑ microsatellite instability / immunohistochemistry

❑ not eligible

❑ other: ……………………………….

**Highest completed education by counselee*:**

❑ no education

❑ primary education

❑ lower secondary education

❑ preparing for vocational education

❑ general and vocational programmes preparing for tertiary education

❑ higher secondary education

❑ tertiary education

*Low = Low: (pre-)primary education or first stage of basic education;

Intermediate-1: lower secondary or second stage of basic education

Intermediate-2: (upper) secondary education

High: tertiary education

**Who initiated discussion of family history?**

❑ Counselee

❑ Physician

❑ Other:…………………………………..

**In which country are counselee and his/her parents born?**

Counselee: ❑ The Netherlands ❑ Other: ……………………………….

Father: ❑ The Netherlands ❑ Other: ……………………………….

Mother: ❑ The Netherlands ❑ Other: ……………………………….
